# Connecting disciplines and decades

**DOI:** 10.1073/pnas.2213953119

**Published:** 2022-10-10

**Authors:** Ilan Kelman

**Affiliations:** ^a^Institute for Risk and Disaster Reduction, University College London, London WC1E 6BT, United Kingdom;; ^b^Institute for Global Health, University College London, London WC1N 1EH, United Kingdom;; ^c^University of Agder, 4630 Kristiansand, Norway

Thank you to the recent“Climate Endgame” perspective (1) for the apt call for more investigation into “bad-to-worst-case scenarios” of human-caused climate change, including possibilities for “worldwide societal collapse or even eventual human extinction.” As researchers, we understandably always promote more research while retaining, as a high priority, new science taken up by policy and practice agendas. As researchers, we also have a duty to ensure that we fully draw on previous work—even before the 1988 date given in the perspective’s (1) second sentence.

The perspective’s (1) core point of needing to know more about “areas characterized by high uncertainties and tail risks” is detailed conceptually, and for policy and practice, in older work, such as “normal accidents” (2) and “high-reliability theory” (3), in addition to publications from volcanology and human ecology from the same era. This science proffers a substantive baseline for the particular example of human-caused climate change within the context of wider, global environmental and social changes.

All this science from across decades and disciplines is further underpinned by numerous discussions in foundational pieces of modern disaster research covering the meanings of “extreme” and how “extreme” disastrous changes might become, for the environment, for humanity, and for their inseparability. *The Environment as Hazard* (4) offered one perspective countered by *Interpretations of Calamity* (5). These books stimulated analyses regarding scales of change (6) examining how everyday disasters tend to affect people more cumulatively over the long term than rarer catastrophes. Thus, it becomes difficult to communicate and act on low-probability, high-consequence incidents, yet they remain important and undervalued (7).

The latter study (7) explicitly refers to human-caused climate change. Also on climate change specifically, see one 1974 analysis (8). An even earlier piece (9), the reactions to it, and the refining of it to overcome the errors meant assessing all risks including low-probability, high-consequence instances.

Seeking to implement and boost the perspective’s (1) call for action could engage with this rich historical and multidisciplinary baseline, considering how few citations in the perspective (1) bear a 20th-century date. We could also integrate philosophical explorations from across centuries and cultures, providing plenty on existentiality, its importance especially when determined or averted by humanity’s collective actions, and how to express these topics and their consequences.

Such material nuances the perspective’s (1) uncritical application of notions such as “tipping points/elements” and “planetary boundaries.” Both have enlightening discussions published and ongoing—along with a 20th-century scientific history—on alternative theories and contrary empirical evidence. As one recent example, see ref. 10 for discussion about ecological tipping points.

The perspective’s (1) ethos and conclusions are apposite and needed, certainly deserving of the repetition provided by the perspective (1) to support continuing scientific investigation for policy and practice. This ethos and these conclusions have long been accepted as truisms and as starting points across many scientific fields and societies.

Thank you for learning from other areas, especially across histories and cultures.
